# Human Placental Hofbauer Cells Maintain an Anti-inflammatory M2 Phenotype despite the Presence of Gestational Diabetes Mellitus

**DOI:** 10.3389/fimmu.2017.00888

**Published:** 2017-07-31

**Authors:** Carolin Schliefsteiner, Miriam Peinhaupt, Susanne Kopp, Jelena Lögl, Ingrid Lang-Olip, Ursula Hiden, Akos Heinemann, Gernot Desoye, Christian Wadsack

**Affiliations:** ^1^Perinatal Research Laboratory, Department of Obstetrics and Gynecology, Medical University of Graz, Graz, Austria; ^2^Department of Experimental and Clinical Pharmacology, Medical University of Graz, Graz, Austria; ^3^Department of Cell Biology, Medical University of Graz, Graz, Austria; ^4^Department of Histology and Embryology, Medical University of Graz, Graz, Austria

**Keywords:** placenta, Hofbauer cells, gestational diabetes, inflammation, macrophage phenotype/polarization

## Abstract

**Background:**

Hofbauer cells (HBCs) are macrophages of the feto-placental unit. Despite the general view that these cells have an anti-inflammatory M2 phenotype, recent studies have claimed that pregnancy pathologies—e.g., gestational diabetes mellitus (GDM)—cause a switch from an M2 to an M1 pro-inflammatory phenotype in HBCs. The pilot-study presented here challenges this claim, showing that HBCs maintain anti-inflammatory properties in spite of the hyperglycemic, low-grade inflammatory environment of GDM.

**Methods:**

HBCs were isolated from placentae of healthy women (*N* = 5) and women with GDM (*N* = 6) diagnosed in the second trimester. FACS was used to measure surface markers associated with either M1 or M2 phenotype on the cells. In addition, placental tissue sections were subjected to immune histochemical imaging to assess the phenotype within the tissue context. Supernatant from control and GDM HBCs was collected at defined time points and used in a multiplex ELISA-on-beads approach to assess secretion of cytokines, chemokines, and growth factors. The effect of HBC cell culture supernatant on placental endothelial activation was investigated.

**Results:**

FACS and immune staining showed that, indeed, M2 markers, such as CD206 and CD209, are increased in HBCs isolated from GDM placentae. Also, the M1 marker CD86 was increased, but only by trend. Secretion of numerous cytokines, chemokines and growth factors was not changed; pro-inflammatory interleukin (IL)-1β and IL-6 release form GDM HBC was increased but not significant. Exposure to GDM HBC supernatant did not induce cell adhesion molecules (VCAM-1, selectins, vascular endothelial-cadherin) in placental endothelial cells compared to supernatant from control HBCs, an induction of intracellular adhesion molecule 1 was observed however.

**Conclusion:**

Our study—although performed in a small set of patients—shows that placental macrophages maintain their anti-inflammatory, tissue remodeling M2 phenotype even in pregnancies affected by gestational diabetes. This consistent phenotype might be important for propagation of maternal tolerance toward the fetus and for protection of the fetus from a low-grade inflammatory environment.

## Introduction

Macrophages represent the first line of defense in numerous human tissues and are crucial to both acute and resolving immune responses. These remarkably plastic cells are able to adapt to their micro-environment in response to various exo- and endogenous stimuli. Therefore, macrophages have been assigned phenotypes called M1 and M2 (1, 2). Macrophages classified as M1 are the so-called classically activated macrophages and are considered to have a pro-inflammatory phenotype. Typical cytokines inducing M1 polarization are, e.g., interferon gamma (IFN-γ) and granulocyte macrophage colony stimulating factor (GM-CSF) but also bacterial endotoxins (lipopolysaccharides) induce and drive the M1 phenotype (3, 4). Macrophages classified as M2 are also referred to as alternatively activated macrophages. They are induced by anti-inflammatory cytokines, such as interleukin (IL)-4, IL-10, and IL-13 (5–7), and glucocorticoids (GCs) (8). Macrophage polarization is reflected by a different repertoire of surface receptors and secreted cytokines [for review see Ref. (9, 10)]. Typical surface molecules on M1 polarized macrophages are CD80, CD86, and IL-1R as well as toll-like receptors. Furthermore, M1 macrophages secrete cytokines of the tumor necrosis factor (TNF) family, IL-1, IL-6, IL-12, and IL-23, and chemokines, e.g., CCL2 (also macrophage chemotactic protein, MCP-1). M2 macrophages express macrophage mannose receptor (MMR/CD206), hemoglobin scavenging receptor (CD163) and CD200R and typically secrete IL-10, interleukin-1 receptor antagonist (IL-1RA), and transforming growth factor β (TGFβ). Some authors also used functional aspects to describe macrophage polarization. Whereas opsonization and phagocytosis of pathogens is the major function of M1 polarized macrophages, M2 macrophages have been described to play a role in immune regulation and tolerance, as well as tissue remodeling, in addition to phagocytic actions.

Categorizing macrophages as M1 or M2 is an oversimplified concept, because of their plasticity and adaptability, and a wide range of intermediates has been described (11). This is also emphasized by the fact that opposed to just one known M1 phenotype, several M2 phenotypes—M2a, M2b, M2c, and M2d—have been described (2, 12). Intriguingly, M2b macrophages share certain properties with M1 macrophages (13).

Placental Hofbauer cells (HBCs) are tissue macrophages of the feto-placental unit and it is now widely accepted that HBCs are of fetal origin. Throughout the first trimester of pregnancy they arise from mesenchymal progenitor cells (14); in the second and third trimester, it is suspected that monocytes are recruited from the fetal circulation and differentiate to HBCs within the placenta (15, 16). HBCs are assumed to move through the placental stroma toward sites where they are needed (17). They possess a very specific morphology characterized by an occurrence of visible granulose vacuoles. A recent study showed that these vacuoles are a result of exposure to and uptake of β-hCG and that this specific phenotype can also be promoted in other macrophage cell lines by stimulation with β-hCG (18). We and others have shown that HBCs have an M2 anti-inflammatory, regulatory phenotype. This phenotype has been underpinned by various studies: HBCs are stimulated by GCs (19) and IL-10 (20); express CD163, CD206, and CD209 (20); and secrete IL-10 and TGFβ (21). Furthermore, DNA methylation profiling of HBCs indicated a programmed M2 phenotype (22). HBCs are implicated in placental vasculogenesis and angiogenesis (23, 24) and we recently demonstrated a regulation of placental endothelial cells by HBC *in vitro* (25). Moreover, HBC are thought to play a role in maternal immunological tolerance against the fetus (26). This indicates a regulatory, tissue remodeling rather than an inflammatory macrophage phenotype. Also, it was shown that even inflammatory pathologies such as chorioamnionitis do not alter HBC phenotype (27). Nevertheless, several studies reported a potential switch toward the pro-inflammatory M1 profile in pregnancies affected by intrauterine infection (28) or diabetes (29).

During pregnancy, maternal metabolic adaptation ensures fetal energy and nutrient supply. This includes the establishment of physiological insulin resistance to form a glucose gradient across the placenta (30, 31). Gestational diabetes mellitus (GDM) occurs if the mother cannot adapt to this insulin resistance. GDM prevalence ranges from 3 to 20% of pregnant women with around 5% in Central Europe (32). Gestational diabetes is associated with a chronic low-grade pro-inflammatory profile in the placenta (33, 34) in which HBCs might play an essential role. Our study aimed to investigate the polarization of human HBCs from normal pregnancies and pregnancies complicated by GDM. In addition, macrophage ability to activate feto-placental endothelial cells was investigated to identify potential functional differences.

## Materials and Methods

### Isolation of HBCs

Placentas were obtained within 20 min after both cesarean sections and vaginal deliveries. Patient characteristics are shown in Table 1. The study was approved by the institutional ethics committee of the Medical University of Graz (27-265 ex 14/15) and all mothers gave written informed consent. Placentas from healthy singleton pregnancies were used as controls. GDM macrophages were isolated from singleton pregnancies when GDM was diagnosed by an oral glucose tolerance test within the second trimester of pregnancy according to ADA criteria (35). Although the study groups were matched for maternal BMI, a predisposing factor for GDM and often considered a confounder in GDM studies (36), groups could not be matched for gestational age (GA, see Table 1). It is common obstetric practice to deliver GDM children a bit premature to avoid complications, such as macrosomia and shoulder dystortia (37–39). However, as placental weight and fetal ponderal index did not differ significantly, one might consider that placenta and children were equally well developed in both groups. The number of HBCs in placenta steadily declines from first trimester to full term, but polarization does not change intensely during this time (40); we, therefore, considered the apparent difference in GA negligible.

**Table 1:** Patient characteristics of women (and their children) included in the study for macrophage isolation.

	Control (*n* = 5)	GDM (*n* = 6)	*p*-Value
Maternal pre-gravid BMI (kg/m^2^)	21.7 ± 2.7	29.7 ± 8.9	ns
Maternal BMI at delivery (kg/m^2^)	27.1 ± 4.0	32.8 ± 7.6	ns
Gestational age (GA) (weeks ± days)	40 ± 4	38 ± 4	0.02*
Placental weight (g)	546.0 ± 43.4	648.0 ± 164.4	ns
Mode of delivery	SP 3, CS 2	SP 2, CS 4	ns
Fetal ponderal index (kg/m^3^)	2.7 ± 0.2	2.7 ± 0.2	ns
Fetal sex	♂3 ♀2	♂4 ♀2	ns

*SP, spontaneous delivery; CS, cesarean section; GDM, gestational diabetes mellitus*.

*Apart from GA, none of the parameters differed between control and GDM subjects. The duration of pregnancy was significantly longer in control pregnancies than those complicated by GDM. This might be explained by the mode of delivery (38); both spontaneously delivered placentae and placentas delivered by CS were used for macrophage isolation—however, in GDM pregnancies, CS is recommended within the standard of care procedure and is often scheduled before 40 weeks of gestations, whereas most children in the control group were delivered at full term (≥40 weeks of gestation). All data are shown as mean ± SD, two-tailed *t*-test was calculated for numerical data, Fisher’s exact test was employed to test significance of categorical data*.

*p < 0.05.

For isolation, the upper layer of maternal membranes was removed from the placenta to avoid contamination with decidual macrophages. The tissue was dissected, finely minced, and stored overnight in PBS buffer. Between 60 and 100 g tissue was used for isolation. The next day, tissue was digested in two steps, first employing trypsin (10×, Gibco) and DNase I (Roche), and second using Collagenase A (Roche) and DNase I. After digestion, cells were applied onto a Percoll gradient (Gibco) and centrifuged at 2,030 × *g* for 30 min, without brake. Macrophages appeared as band between the 30 and 35% Percoll layers. Cells were aspirated from the gradient and negative immune selection with magnetic beads (Dynabeads anti-goat IgG, Invitrogen) and antibodies against CD10 (abcam) and EGFR (NeoMarkers) was used to further purify the cells. After immune selection, cells were counted and seeded in macrophage medium [macrophage medium (MaM), ScienCell] supplemented with 5% FCS and macrophage growth supplements (ScienCell) at a density of 1 × 10^6^ cells/ml. Cells were cultivated at 21% oxygen, 37°C; quality control was carried out by loading HBCs with Ac-Dil-LDL after 2 days and monitoring fluorescence in the live cells; and immune cytochemistry (ICC) after 7 days on fixed cells.

### Western Blot

Hofbauer cells isolated from control placentas were plated at a density of 1 × 10^6^ cells/ml in 6-well culture dishes (3 ml total volume). On day 3 post-isolation, cells were serum-starved for 12 h and thereafter switched to complete MaM containing either 25 mM d-glucose (Sigma) to mimic maternal and fetal hyperglycemia, 10 nM Insulin (Calbiochem) to mimic fetal hyperinsulinemia in response to maternal GDM, or a combination of both. Equimolar l-glucose (Sigma) was used as osmatic control, an untreated control grown in MaM only was included. Cells were cultivated for 72 h, receiving treatment every 24 h. Cells were harvested and lysed using RIPA buffer. Protein content was measured using bichinonic acid method (BCA assay, Pierce). 7.5 µg of protein was subjected to electrophoresis (4–20% Mini-Protean TGX gels, Biorad) and blotted onto nitrocellulose membranes (Trans-Blot Turbo System, Biorad). Membranes were incubated with antibodies against CD163 (Thermo Scientific), CD86 and CD209 (both NovusBio) and β-Actin as loading control (abcam); secondary antibodies against mouse and rabbit IgG were from Biorad. Detection was carried out using West Femto ECL substrate (Pierce) on a ChemiDoc XRS system (Biorad).

### Cytokine Multiplex and ELISA Validation

Secretion of pro- and anti-inflammatory cytokines, chemokines, and growth factors from placental macrophages was assessed using a multiplex ELISA-on-beads approach. Control (*N* = 5) and diabetic macrophages (*N* = 6) were cultivated in MaM up to 4 days, a time point taken every 24 h. Due to the low levels of cytokines in the supernatant, samples were concentrated fourfold using Amicon Ultra centrifugal units (3kD MwCo, Millipore), to be in the multiplex assay range. A panel of 23 factors of interest (see Table 2) was obtained as customized panel (Aimplex, YSL Bioprocess Development Co.) and the multiplex experiment was carried out according to the manufacturer’s instructions. Bead signals were quantified using a FACS Calibur instrument (Becton Dickinson) and FlowCytomixPro software (eBioscience) was used for calculation of standard curves and sample concentrations. For data normalization, total protein of the supernatant was assessed using BCA Kit and cytokine levels where normalized to total protein levels accounting for eventually divergent concentration of volume. ELISAs used for validation were carried out according to assay manuals. ELISAs used were IL-1β and IL-13 (both eBioscience), IL-1RA, IL-6, IL-8, IL-12, macrophage colony stimulating factor (M-CSF), TNFα, intracellular adhesion molecule 1 (ICAM-1), and vascular endothelial growth factor (VEGF) (all Peprotech) and TGFβ (eBioscience).

**Table 2:** Macrophage yield from control and diabetic placental tissue.

	First cell count	Second cell count	*p*-Value
		
	10^6^ cells/g tissue wet weight	
Control	3.2 ± 1.8	1.8 ± 0.7	ns
Diabetic	3.0 ± 1.0	1.7 ± 0.4	ns

*Average yield was calculated from *n* = 7 macrophage isolations per group, and with respect to the wet weight (in grams) of tissue used for each respective isolation. First cell count is performed before immune purification, the second cell count afterward. All data are shown as mean ± SD, two-tailed *t*-test*.

### Fluorescence-Assisted Cell Sorting

Macrophages cultured for 7 days were carefully harvested using Dispase enzyme solution and gentle scraping. Cells were counted and a minimum of 3 × 10^5^ cells were used for each FACS staining. Fc receptors were blocked in 3% BSA in 1× HBSS (Gibco) for 10 min. As this study focused on membrane proteins, permeabilization and fixation of cells were omitted. Instead, cells were re-suspended in PBS and incubated with the respective antibodies for 30 min. Antibodies used for FACS were αCD163-APC and αCD90-PE (both Biolegend), CD11b-V450, CD11c-PE, CD40-FITC, CD45-PE, CD209-Cy5.5, CD80-V450, CD86-V450, CD146-PE, and CD206-FITC (all BD Pharmingen). Unstained cells were used as control, as well as an IgG control. Cells were washed twice after antibody incubation and re-suspended in PBS for counting. Cell sorting was performed on a LSR-II instrument (BD Bioscience) using FACSDiva8 acquisition and analysis software. Cells were gated in three steps; first, cells were discriminated by size employing forward and side scatter (FSC and SSC, respectively). Second, doublets were removed by pulse-geometry gating the area and height of FSC. Lastly, the fluorescence signal of cells positive for respective markers was gated directly, plotting it against the SSC area.

### Immune Cytochemistry

Immune cytochemistry was carried out on 4-well glass chamber slides (Lab-Tek) using isotype controls and reagents from Dako. Briefly, cells were washed twice with HBSS and fixed with ice cold acetone. Antibodies were diluted to working concentration using Dako antibody diluent and cells were incubated with primary antibodies for 30 min. Cells were subsequently incubated with Dako antibody enhancer for 10 min. After a washing step, cells were incubated with large HRP polymer solution and AEC chromogen solution, for 20 and 10 min, respectively. Cells were counterstained with haemalaun solution and mounted with glycerin. Antibodies used were αCD163 (Thermo Scientific), αCD90 (Dianova), αCD68 (Dako), αCD11b (abcam), αCD11c (abcam), α CD14 (BD Pharmingen), αCD209 (Novus), and α mouse IgG (Dako) as isotype control. For quantification, pictures from four different HBC isolations per group were counted and positive cells were expressed as percentage relative to the total number of cells on each picture. To avoid investigator bias, all countings were done by one researcher alone.

### Immune Histochemistry

Tissue sections of 5 µm thickness were cut from paraffin-embedded placental tissue (taken from the fetal side and a central region of the placental disk) and mounted onto glass slides. After eliminating the paraffin using xylene and rehydrating the tissue in an ethanol dilution series, antigen retrieval was carried out using 1 mM EDTA. For immune histochemical staining, the UltraVision LP detection system by Dako was employed: tissue was incubated for 15 min in Hydrogen Peroxide Block and washed four times in TBE buffer, followed by 5 min incubation with UltraV Block. Samples were subsequently incubated with the primary antibody, followed by incubation with primary antibody enhancer, for 30 and 10 min, respectively. Antibodies used were αCD163 (Pierce), αCD68 (Dako), αCD40 (Abcam), αCD11b (Abcam), and αCD11c (Abcam). Samples were washed again in TBE and HRP polymer was applied for 30 min. After another washing step, samples were incubated with AEC chromogen solution for 10 min. Samples were counterstained with haemalaun and mounted using glycerin. Spleen and lymph node tissue, embedded in paraffin, were prepared and stained in the same manner and used as positive control, as these tissues are supposed to express all investigated markers according to the Human Protein Atlas tissue bank. Photographs were taken using Zeiss AxioVision software v8.0 on an Olympus BX53 light microscope with an AxioCam MRc5 (Zeiss). For quantification, positive cells on the pictures were counted by one single histologist with the help of the Visiopharm Stereotopix™ software platform to avoid intra-individual bias. Pictures from four placentas per group were taken, and seven to ten different sites from each placenta were used for quantification. Positive cells were expressed relative to CD163, which proved to be the most consistent marker of HBCs.

### Endothelial Cell Activation

Human placental arterial endothelial cells (pAECs) were isolated as described before (41). HBCs isolated from control and GDM placentas were cultivated for 6 days in MaM (1 × 10^6^ cells/ml medium), then supernatant was collected. To obtain conditioned medium (CM), equal parts of three different HBC isolations were mixed1:1 with endothelial basal medium (EBM, Lonza) without growth supplements and only 2% FCS. pAECs were exposed to macrophage CM from control and GDM HBCs to unconditioned MaM as negative control l. In addition, a positive control was made from EBM containing a cytokine mix of TNFα (1.5 ng/ml), IL-1β (0.2 ng/ml), and IL-6 (1 ng/ml). After 48 h treatment (37°C, 5% CO_2_, 12% O_2_), pAEC were harvested and lysed in cell lysis buffer (Raybiotech). A custom Quantibody ELISA array (Raybiotech) was used to detect ICAM-1, vascular adhesion molecule 1 (VCAM-1), P-selectin, and E-selectin, as well as vascular endothelial (VE)-cadherin and M-CSF in parallel in the lysates. The array way prepared according to the manufacturer’s instructions and fluorescence scanning was performed on an Agilent Microarray scanner G2565CA. Data extraction was done using GenePix Pro v6.0 and analysis was carried out using a software tool for MS Excel provided by the manufacturer.

### Statistical Analysis

GraphPad Prism 7 was used for all statistical calculations and plotting of graphs. Shapiro–Wilk test was used to test for normal distribution. For comparison of two groups, *t*-test was used. One-way ANOVA was performed, if more than two groups/time points were compared. In one-way ANOVA, Sidaks *post hoc* test was used to account for multiple comparisons. Kruskal–Wallis test with Dunns test for multiple comparisons was used instead of ANOVA if normality test failed. *p*-Values below 0.05 were considered statistically significant. For *post hoc* power analysis, G*Power 3.1 software (available from the University of Duesseldorf, Germany) was used.

## Results

### *In Vivo*, M1 and M2 Macrophage Subsets Are Present Independent of Diabetes

**Figure 1:**
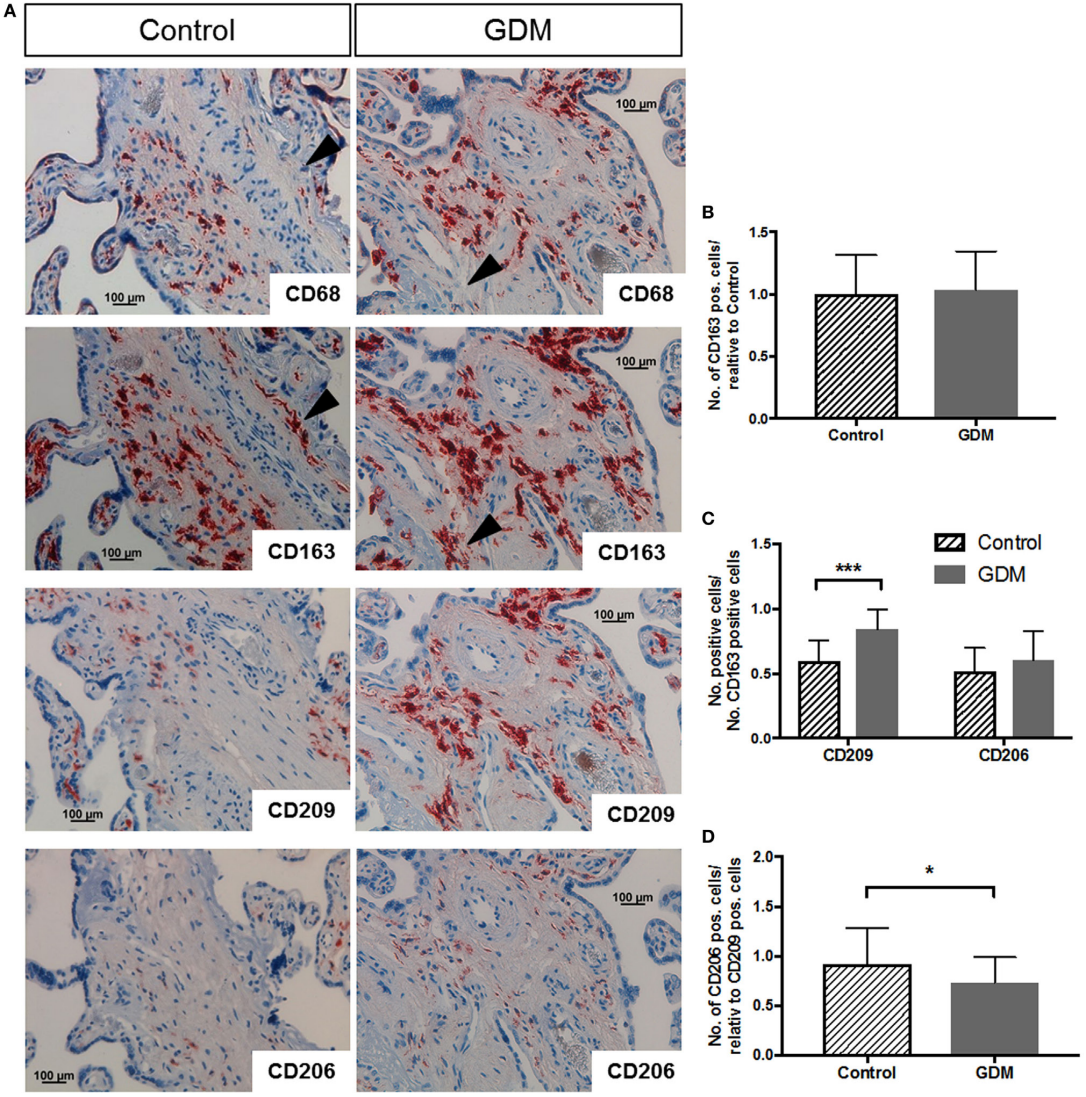
Immune histochemical assessment of M2 macrophage markers in placental tissue. **(A)** Immune histochemical staining against CD68 (top), CD163 (second from top), CD209 (next-to-bottom), and CD206 (bottom) in control (left hand side) and gestational diabetes mellitus (GDM) (right-hand side) placenta. Black arrowheads in CD68 and CD163 pictures indicate regions of (i) absence of CD68 but (ii) presence of CD163 staining in the same region, underpinning that CD68 is not the best marker for Hofbauer cell (HBC) detection. Images representative of serial sections from four different placentas per group are shown. **(B)** Quantification of CD163-positive HBC in control and GDM placenta. **(C)** Quantification of CD209 and CD206 positive cells in control and GDM placenta relative to the number of CD163 positive cells. An increase in CD209 positive cells in GDM tissue was observed. **(D)** Number of CD206 positive cells relative to CD209 positive cells. A decrease in the number of CD206 positive cells in GDM was observed. All data in **(B–D)** are presented as mean ± SD; *t*-test **(B,D)** and one-way ANOVA **(C)**.

Immunohistochemistry was used to characterize macrophages in placental tissue. Markers for M1 and M2 polarization were investigated in serial tissue sections. Although suggested as a pan-macrophage marker, CD68 staining was limited in placenta (Figure 1A, top panel), and CD163 (Figure 1A, second from top) appears to be more abundant and a more reliable HBC marker. On serial sections of placental tissue, we observed regions were CD68 staining was absent, but clearly a staining for CD163 was present; examples of such regions are indicated by the black arrow heads in Figure 1A. Quantification of CD68 did not show any difference in number of positive cells between control and GDM placenta in absolute quantification and also in quantification relative to CD163 (data not shown). Specifically, CD163 positive cells were found in the villous stroma of control and GDM placentas (Figure 1A, second from top). With respect to distribution, independent of the presence of GDM, CD163 positive cells were present in stem villi, intermediate and terminal villi, and positioned with in the stroma, but distant from vessel walls and vessel-surrounding connective tissue. Counting the numbers of CD163 and expressing them relative to control (i.e., control = 100%), we found similar numbers of CD163 positive cells in control and GDM tissue (Figure 1B). CD163 was used in quantification of IHC to account for the overall number of HBCs, and quantification of M2 markers CD209 and CD206 (Figure 1A, center panels) was performed relative to CD163. We observed a significant increase of CD209 positive cells in GDM placenta (*p* < 0.001; Figure 1C), and a slight but non-significant increase in CD206. However, calculating the ratio of CD206 to CD209 positive cells, we observed a decreased number of CD206 positive cells in GDM placenta (Figure 1D).

**Figure 2:**
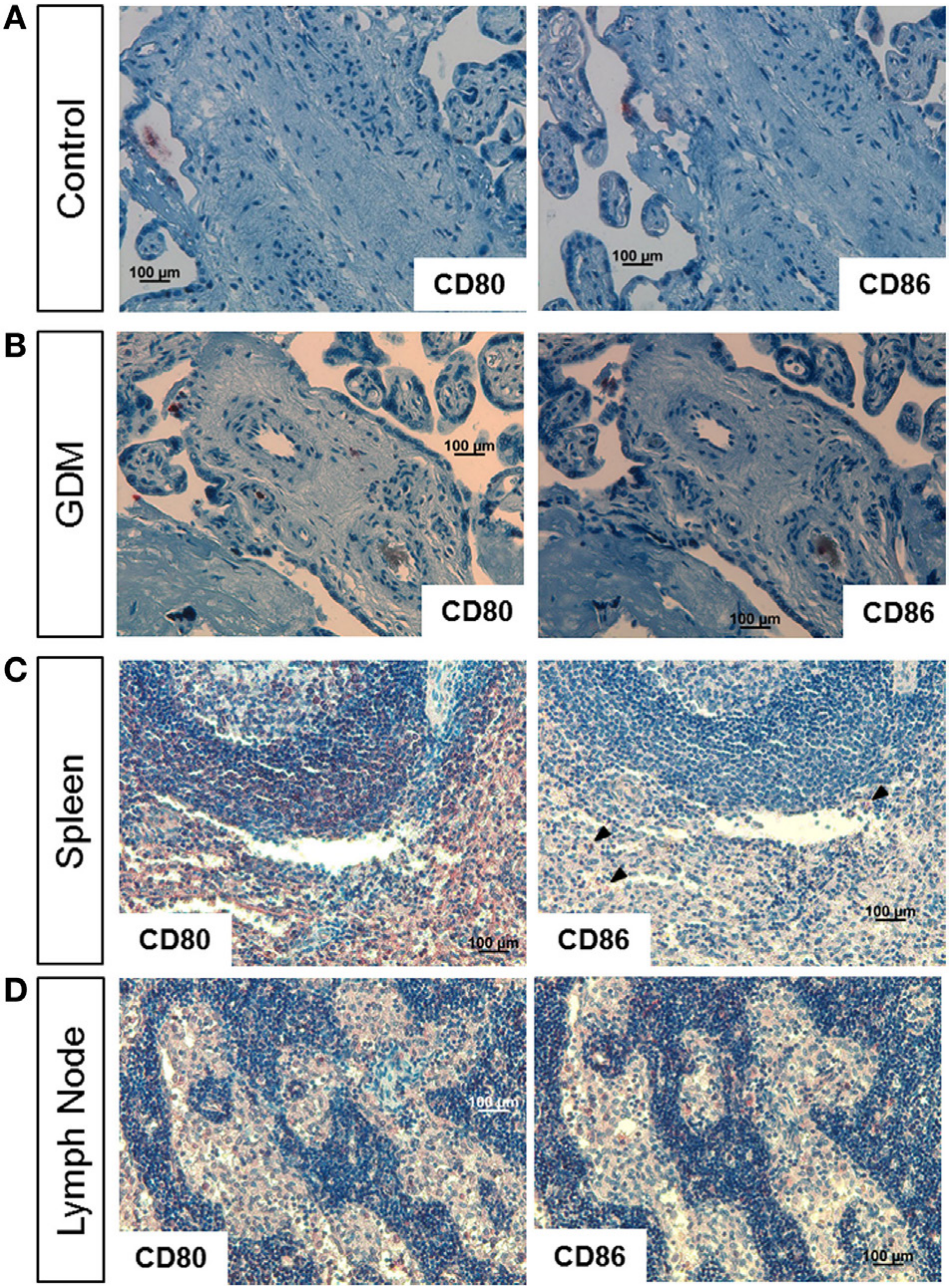
Immune histochemical assessment of M1 macrophage markers in placental tissue. **(A)** Staining of control placental tissue against CD80 (left) and CD86 (right). **(B)** Staining of gestational diabetes mellitus (GDM) placental tissue against CD80 (left) and CD86 (right). **(C)** Staining of human spleen against CD80 (left) and CD86 (right, arrow heads indicate positive cells). **(D)** Staining of human lymph node against CD80 (left) and CD86 (right). Pictures are representative of serial sections from four different placentas per group; spleen and lymph node tissue served as positive controls.

As M1 markers, we used CD80 and CD86; spleen and lymph node tissue served as positive control. In control placenta (Figure 2A, left), no specific staining against CD80 was observed. Also almost no CD86 positive cells were present (Figure 2A, right). In GDM placenta, a very limited number of cells showed CD80 (Figure 2B, left) and CD86 (Figure 2B, right) staining. In spleen (Figure 2C) and lymph node (Figure 2D), which were used as positive control tissues, a much higher number of positive cells were observed.

In addition, we also tested the M1 marker CD40 (Figure S1A in Supplementary Material) and CD11b (Figure S1B in Supplementary Material) in conjunction with CD11c (Figure S1C in Supplementary Material). No specific staining against these M1 markers was observed in control placenta (left panel) as well as GDM placenta (second from left). In the positive controls, spleen and lymph node, staining against these markers was observed.

In line with the slight differences in macrophage marker expression between normal and GDM placentas, also total macrophage cell number was not altered by GDM. Previous data suggest that inflammatory disease of the placenta leads to an infiltration of tissue with HBCs. This has been stated not only for villitis of unknown etiology but also for GDM (42, 43). Therefore, we calculated the yield of macrophages relative to the wet weight of tissue used for isolation and found that total macrophage cell number between control and diabetic isolations did not differ (Table 2). One might also take total placental weight before isolation into account; however, placental weight was not significantly different between control and GDM subjects (Table 1).

### Expression of M1 and M2 Markers Were Similar in Isolated HBC of Control and GDM Placentas

**Figure 3:**
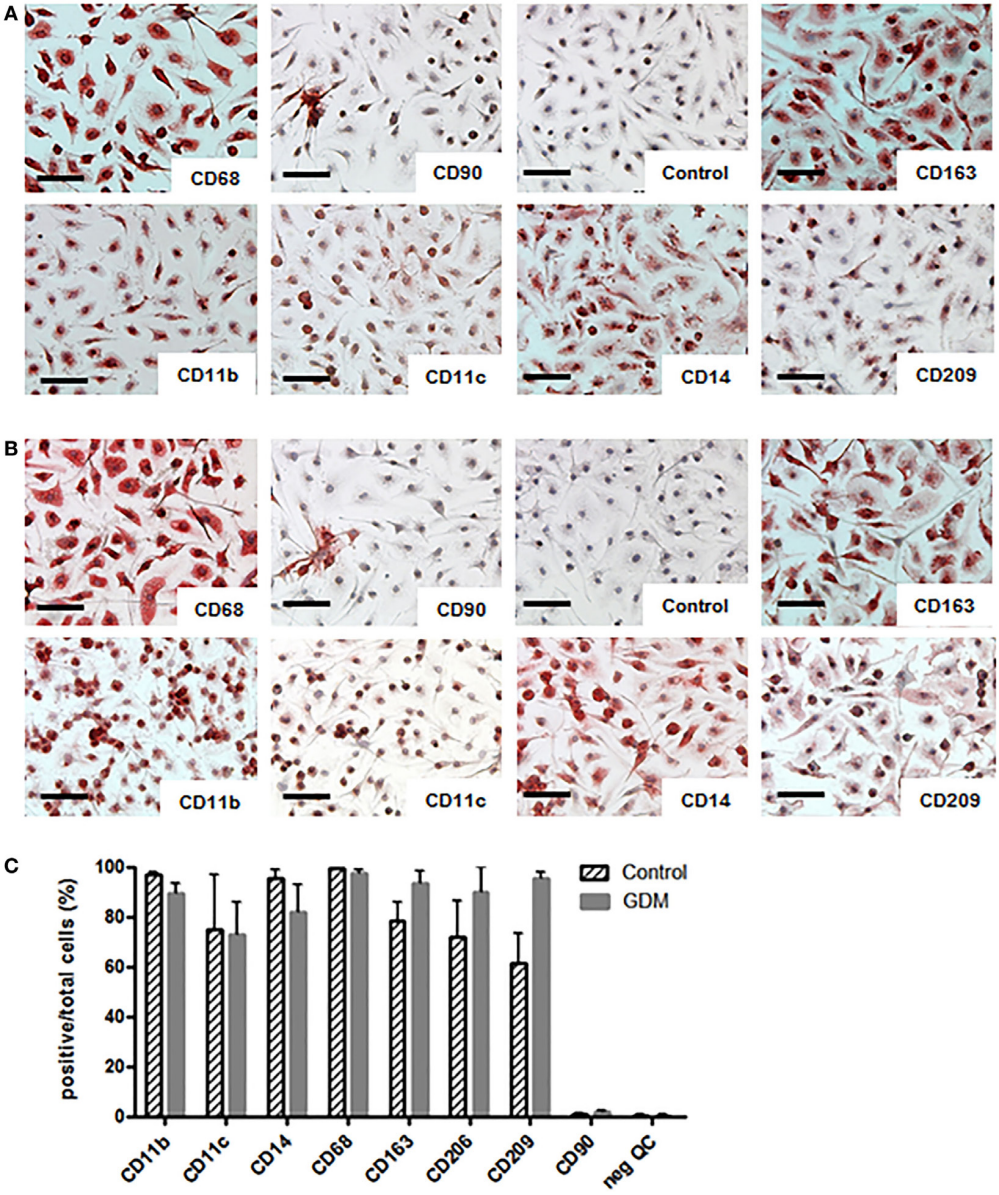
Immune cytochemistry (ICC) for M1 and M2 markers on isolated Hofbauer cells (HBCs). **(A)** HBCs isolated from human placenta after normal pregnancy; **(B)** HBCs isolated from human placenta of gestational diabetes mellitus (GDM) pregnancy. CD68 was used as pan-macrophage marker independent of M1/M2 polarization. CD90 was used to staining contamination with fibroblasts. Anti-mouse-IgG isotype control served as negative control. CD163 and CD209 were used as markers of the M2 phenotype. CD11b in combination with CD11c are markers for M1 macrophages as well as CD14 Images shown are representative of four experiments per group. Scale bar = 200 μm. **(C)** Quantification of ICC; positive cells for each marker were stated relative to the total number of cells; all data are shown as mean ± SD, one-way ANOVA.

The purity of isolated HBCs was confirmed by positive staining for CD163 and CD68 and negative staining for CD90 (fibroblast marker) in immunohistochemistry. CD14 and CD209 were used as markers for M2 polarization, and CD11b together with CD11c as markers for M1 macrophages. We found that HBCs, independent of gestational diabetes, expressed M2 surface markers CD163, CD14, and CD209 (Figure 3A/upper panel: control HBC, Figure 3B/lower panel: diabetic HBC). In addition, HBCs expressed CD11b and also CD11c, which are associated with a pro-inflammatory M1 phenotype and the pan-macrophage marker CD68. ICC was also quantified, counting positive cells relative to total cells (Figure 3C); no significant changes for either M1 or M2 marker expression between control and GDM HBCs were observed, although CD209 appeared slightly increased in GDM. No relevant contamination with fibroblasts (CD90 staining) was observed.

**Figure 4:**
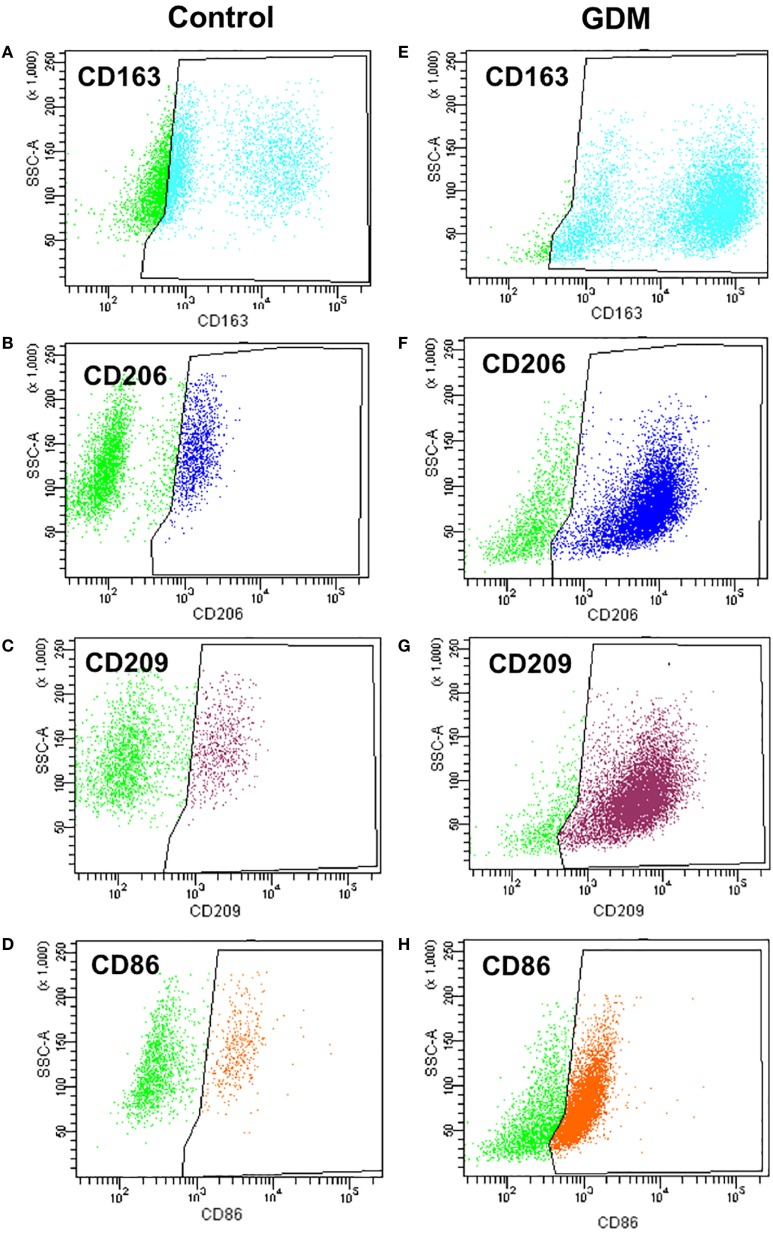
Flow cytometry analysis of M1 and M2 markers in control and gestational diabetes mellitus (GDM)-Hofbauer cell (HBC). **(A,E)** CD163 positive population size was similar in HBCs isolated from control **(A)** and GDM **(E)** placenta. **(B,F)** HBCs isolated from GDM placenta **(F)** constitute a larger population positive for the M2 marker CD206 than control HBC **(B)**. **(C,G)** HBCs isolated from GDM placenta **(G)** constitute a larger population positive for the M2 marker CD209 than control HBC **(C)**. **(D,H)** GDM HBCs tended to have a larger population of CD86 positive cells, representing presence of either M2b or M1 macrophages. *N* = 4 isolations/group were used for FACS analysis, one representative experiment shown.

For a more accurate quantification, the presence of surface markers was also assessed on isolated primary HBCs by flow cytometry. FACS revealed that CD163—the most prominent resident macrophage marker—was evenly distributed between control and diabetic HBC (Figures 4A,E; Table 3). Other scavenger receptors, such as M2 markers CD206 (Figures 4B,F) and CD209 (Figures 4C,G), were indeed significantly increased on HBCs isolated from diabetic placentae (Table 3; *p* = 0.03 and *p* < 0.001, respectively). CD11b and CD11c were found both on control and diabetic HBC and did not differ in the percentage of their population (Table 3). Also the ratio of CD11b positive to CD11c positive cells, which was proposed as a measure of a shift toward M1 macrophage populations (44), was unchanged. Moreover, in GDM-HBC a higher percentage of cells was positive for M1 markers CD80 and CD40, but these increases did not reach significance (Table 3); however, the M1 marker CD86 (Figures 4D,H) showed a trend toward increased levels in GDM (*p* = 0.08; Table 3).

**Table 3:** Flow cytometry analysis of M1 and M2 population markers in control and gestational diabetes mellitus (GDM) Hofbauer cell.

	Surface marker	Control (*n* = 4)	GDM (*n* = 4)	*p*-Value
		
		% Population	
M2	CD163	85.0 ± 21.3	97.3 ± 3.3	ns
	CD206	42.8 ± 19.6	85.0 ± 7.6	0.033
	CD209	15.3 ± 9.7	87.4 ± 11.8	<0.001

M1	CD11b	62.1 ± 32.8	87.8 ± 5.0	ns
	CD11c	72.8 ± 23.9	97.4 ± 2.6	ns
	CD11b/CD11c ratio	0.8 ± 0.4	0.9 ± 0.1	ns
	CD80	3.9 ± 2.1	6.8 ± 5.9	ns
	CD86	31.3 ± 26.4	64.5 ± 8.2	0.08
	CD40	19.3 ± 4.6	44.7 ± 30.9	ns

*Population data of n = 4 isolations per group was pooled for analysis, all data are shown as mean ± SD, two-tailed t-test*.

**Figure 5:**
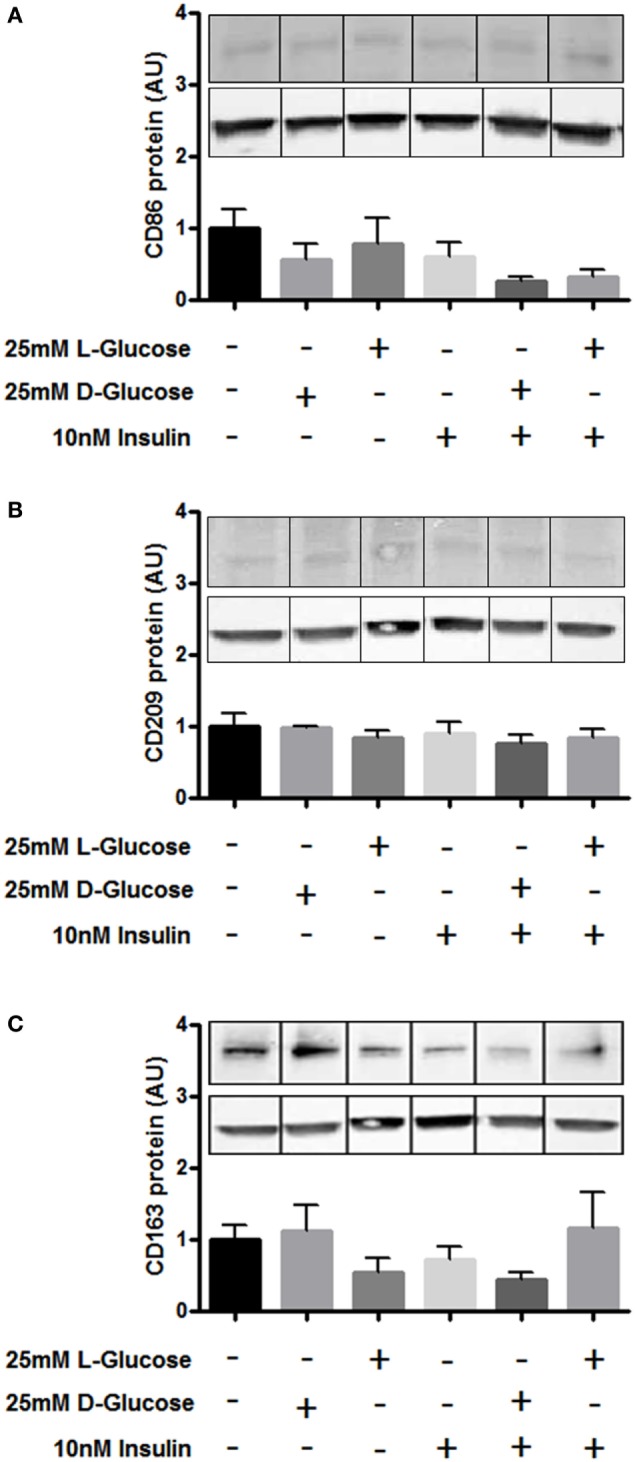
Surface marker expression on Hofbauer cells (HBCs) in response to hyperglycemia and insulin. **(A)** Protein level of M1 marker CD86 after exposure to glucose, insulin, and the combination of both. **(B)** Protein level of M2 marker CD209 after exposure to glucose, insulin, and the combination of both. **(C)** Protein level of HBC marker CD163 was not affected by exposure to glucose, insulin, and the combination of both. l-Glucose was used as osmotic control, cells cultivated in macrophage medium only were used as untreated control. Bar charts: pool of three individual experiments, one-way ANOVA; mean ± SEM. One out of three representative Western Blots shown. Western Blot images were cropped as gels were loaded with side-by-side duplicates of each treatment. β-Actin was used as loading control for normalization.

Furthermore, we investigated whether hyperglycemia or hyperinsulinemia induce or alter HBCs polarization *in vitro*. HBCs isolated after normal pregnancies were exposed to 25 mM d-glucose, 10 nM insulin, or a combination of both for 72 h. l-Glucose was used for control conditions. Western Blot analysis revealed that none of the treatments altered protein levels of CD163, CD209, and CD86 (Figures 5A–C).

### Cytokine Release from HBCs Remains Unaffected by Gestational Diabetes

**Figure 6:**
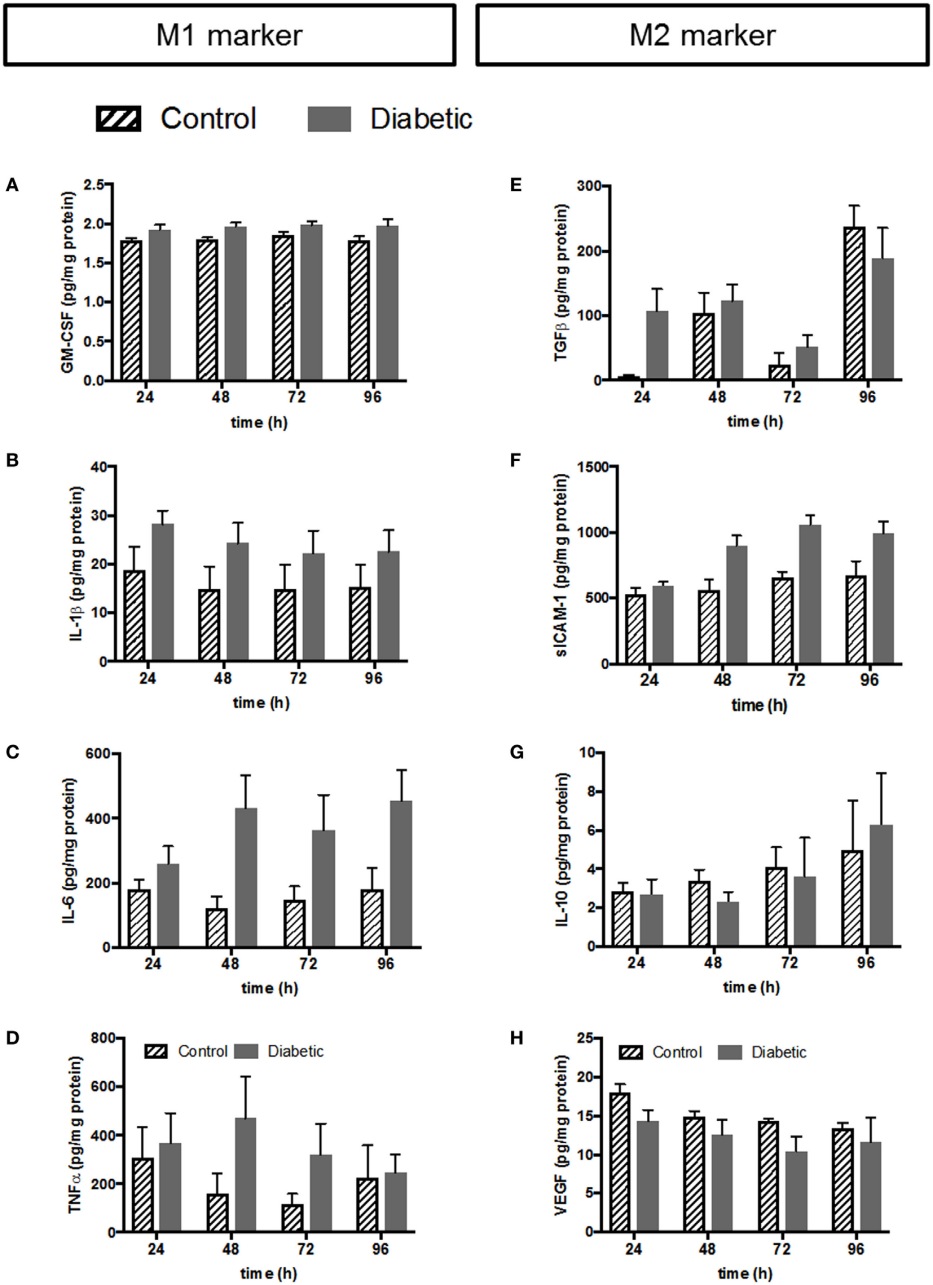
Validation of selected cytokines and growth factors from multiplex by conventional ELISA. Left panel: cytokines associated with the M1 phenotype; **(A)** ELISA against GM-CSF. **(B)** ELISA against IL-1β. **(C)** ELISA against IL-6. **(D)** ELISA against TNFα. Right panel: cytokines and growth factors associated with the M2 phenotype; **(E)** ELISA against TGFβ. **(F)** ELISA against sICAM-1. (G) ELISA against IL-10. **(H)** ELISA against VEGF. Pooled data: *n* = 5 control HBCs, *n* = 6 GDM HBCs, Kruskal–Wallis test (ANOVA on ranks), mean±SEM.

Using Multiplex ELISA-on-bead technology, we assessed the secretion of 23 cytokines, chemokines and growth factors by HBC into the medium after 24, 48, 72, and 96 h. IL-4, IFNγ, IL-1α, and epithelial growth factor were below the detection limit of the assay. The remaining 19 compounds could be quantified and were normalized to total protein of the respective supernatants. The results revealed only slight differences between normal and diabetic HBCs with respect to cytokine secretion (all data summarized in Table S1 in Supplementary Material). Results were further validated by ELISA (Figure 6).

The left panel of Figure 6 shows release of cytokines and chemokines associated with the M1 phenotype from HBC. GM-CSF release was only borderline difference between normal and GDM HBCs as measured by multiplex (Table S1 in Supplementary Material) and ELISA confirmed that there is no different in GM-CSF secretion (Figure 6A). Noteworthy, although GM-CSF is generally associated with the M1 phenotype, some studies have indicated that macrophages GM-CSF aids wound healing and tissue remodeling; therefore GM-CSF has also been linked with the M2b phenotype (45). IL-1β release from GDM-HBC was consistently elevated over all time points (Figure 6B; plus ~25%) compared to control HBC. However, this increase did not reach statistical significance, neither in multiplex (Table S1 in Supplementary Material) nor in ELISA. The same observation was made for IL-6 (Figure 6C; plus ~50%). Also, the release of the classic M1 cytokine TNFα was unchanged between normal and diabetic HBCs, although release from GDM HBC was higher at 48 and 72 h time points (Figure 6D).

Release of cytokines associated with the M2 phenotype is shown in the right panel of Figure 6. Release of TGFβ—a hallmark cytokine of the M2 phenotype—was similar from HBC independent of diabetes. Only at the 24 h time point, TGFβ release tended to be higher (+95%, *p* = 0.06) in GDM-HBC (Figure 6E). Notably, TGFβ was only assessed by ELISA as binding to the TGFβ epitope requires acidification of samples which precluded the use for the multiplex approach. Soluble ICAM-1 (sICAM-1) antagonizes the effects of ICAM-1/LFA-1 signaling and promotes angiogenesis (46, 47), so it can be considered an M2 marker, although it has to be noted that it is not a classical phenotypic marker of either M1 or M2 macrophages. sICAM-1 was significantly increased in the multiplex at 48 h (Table S1 in Supplementary Material); ELISA showed that although the increase in GDM persisted at all time points, it was non-significant (Figure 6F). Moreover, release of IL-10 from control and GDM HBC was not altered (Figure 6G). Levels of IL-12 were not changed between control and GDM HBC (Table S1 in Supplementary Material) and much lower than levels of IL-10 in multiplex (Table S1 in Supplementary Material), yielding IL-10^hi^/IL-12^low^ macrophages, which are considered anti-inflammatory (44). Levels of IL-12 could not even be validated by ELISA because of limit of detection in conventional ELISA. Finally, VEGF release between control and GDM-HBC was not significantly different (Figure 6H). VEGF is considered a pro-angiogenic factor associated with the tissue remodeling phenotype of M2 macrophages.

In addition to the parameters in Figure 5, also other proteins have been validated by conventional ELISA. Like IL-12, IL-13 validation failed due to the detection limit of the ELISA. IL-8 was validated and unchanged between control and GDM HBC; as IL-8 is an ambiguous cytokine involved in neutrophil attraction (an M1 like feature) but also pro-angiogenic (an M2 like feature) it was not included in Figure 5. Also, secretion of macrophage chemoattractant protein (MCP-1), an M1 marker, and IL-1RA, an M2 marker, was not significantly different between control and GDM HBCs.

### Endothelial Cell Activation by HBC Is Not Altered by GDM

Cross talk between tissue macrophages and endothelial cells is a well-established phenomenon, especially in vascular dysfunction and atherosclerosis (48). GDM is known to cause endothelial dysfunction in mother, placenta, and fetus (49, 50). Therefore, we investigated placental endothelial cell activation by HBCs. Macrophage CM from control and GDM HBCs was used to treat primary pAECs. In addition, as a positive control, cells were treated with a cytokine cocktail containing TNFα, IL-1β, and IL-6, which were increased not only in GDM as found in our multiplex approach but also in other studies (51). Unconditioned medium served as negative control. After incubation with CM for 48 h, cells were lysed and lysates were used in a multiplexed, fluorimetric ELISA detecting ICAM-1, VCAM-1, E-selectin, P-selectin, VE-cadherin, and M-CSF.

**Figure 7:**
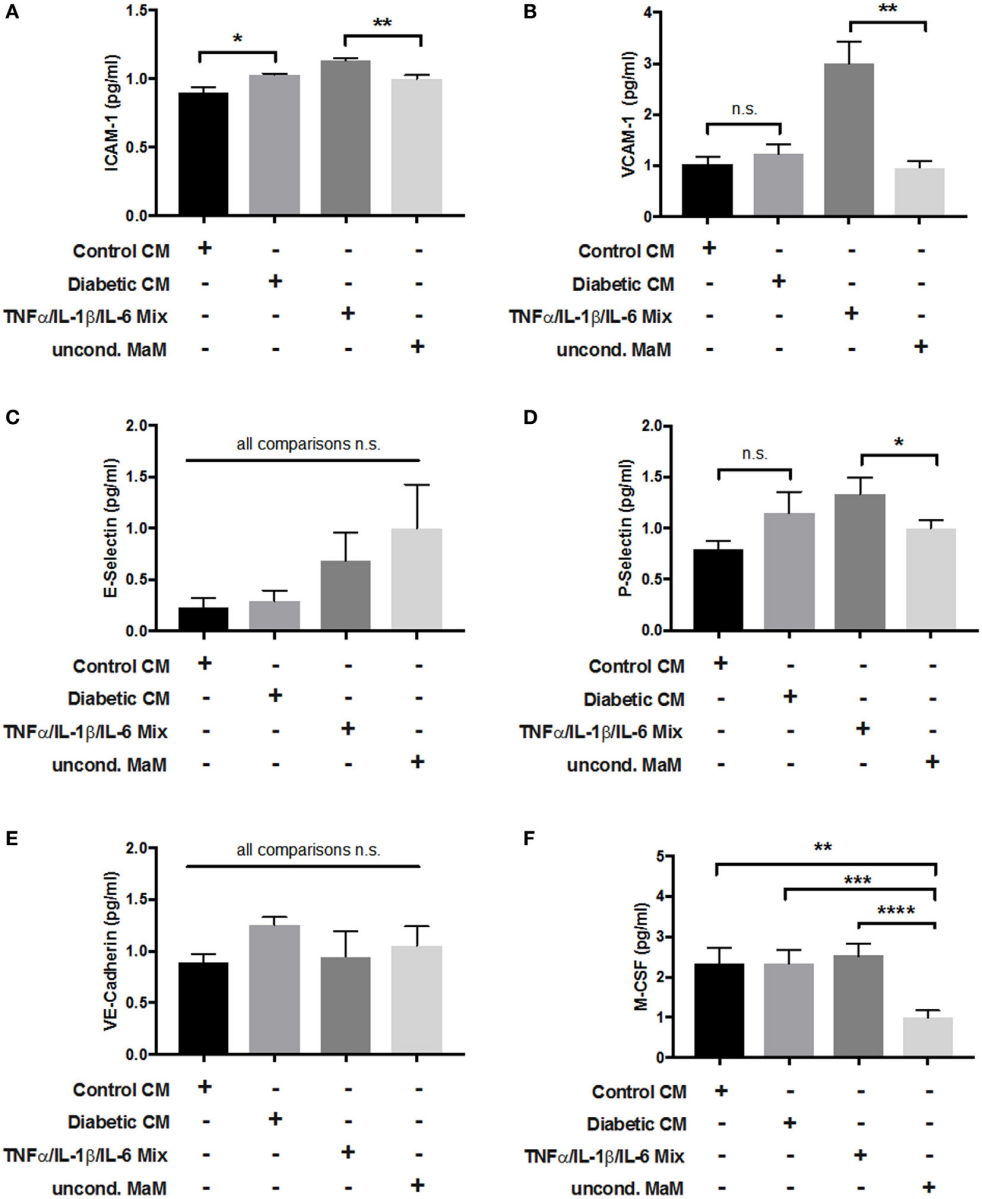
Adhesion molecule expression in endothelial cells in response to stimulation with macrophage-conditioned medium (CM). Placental arterial endothelial cells (pAECs) were incubated with macrophage CM from control and diabetic cells, and appropriate positive [tumor necrosis factor α (TNFα)/interleukin (IL)-1β/IL-6 mix] and negative controls [uncond. macrophage medium (MaM)]. Production of intracellular adhesion molecule 1 (ICAM-1) **(A)**, VCAM-1 **(B)**, E-selectin **(C)**, P-selectin **(D)**, vascular endothelial (VE)-cadherin **(E)**, and macrophage colony stimulating factor (M-CSF) **(F)** in response to treatment was measured. Pools of supernatant from three macrophage isolations were used to prepare CM; the experiment was performed with *n* = 3 different primary pAEC isolations. One-way ANOVA. Data are shown as mean ± SEM.

Intracellular adhesion molecule 1 protein levels in endothelial cells were increased upon incubation with GDM CM when compared to control CM (+12%, *p* < 0.05). Also, the cytokine mix positive control induced EC activation (Figure 7A). VCAM-1 protein was only induced by the cytokine cocktail (threefold, *p* < 0.001), but no other treatment (Figure 7B). E-selectin protein levels were not changed by any treatment (Figure 7C). P-selectin, similar to VCAM-1, was only induced by the cytokine mix (+25%, *p* < 0.05; Figure 7D). VE-cadherin protein expression was unchanged by either treatment (Figure 7E). M-CSF levels were increased about twofold by Ctr-CM (*p* < 0.01), GDM–CM (*p* < 0.001), and cytokine cocktail (*p* < 0.0001) compared to unconditioned medium (Figure 7F).

## Discussion

Gestational diabetes mellitus is a frequent metabolic disorder in pregnant women and is described as any situation of glucose intolerance with onset or first detection in pregnancy. The human placenta is also sensitive to the maternal hyperglycemic milieu and responses with adaptive changes of structure and function. In this study, we asked the question whether placental HBCs—known to be M2 polarized and, therefore, characterized as anti-inflammatory—undergo a switch to pro-inflammatory M1 macrophages in the diabetic environment of GDM. To address this hypothesis, we considered (i) phenotypic cell surface markers on isolated cells and in embedded tissue, (ii) cytokine secretion patterns, and (iii) macrophage-endothelium cross talk. Our findings demonstrate that placental HBCs maintain their M2 phenotype despite the pro-inflammatory, hyperglycemic environment of GDM; however, the M2 subtype seems altered by GDM.

**Figure 8:**
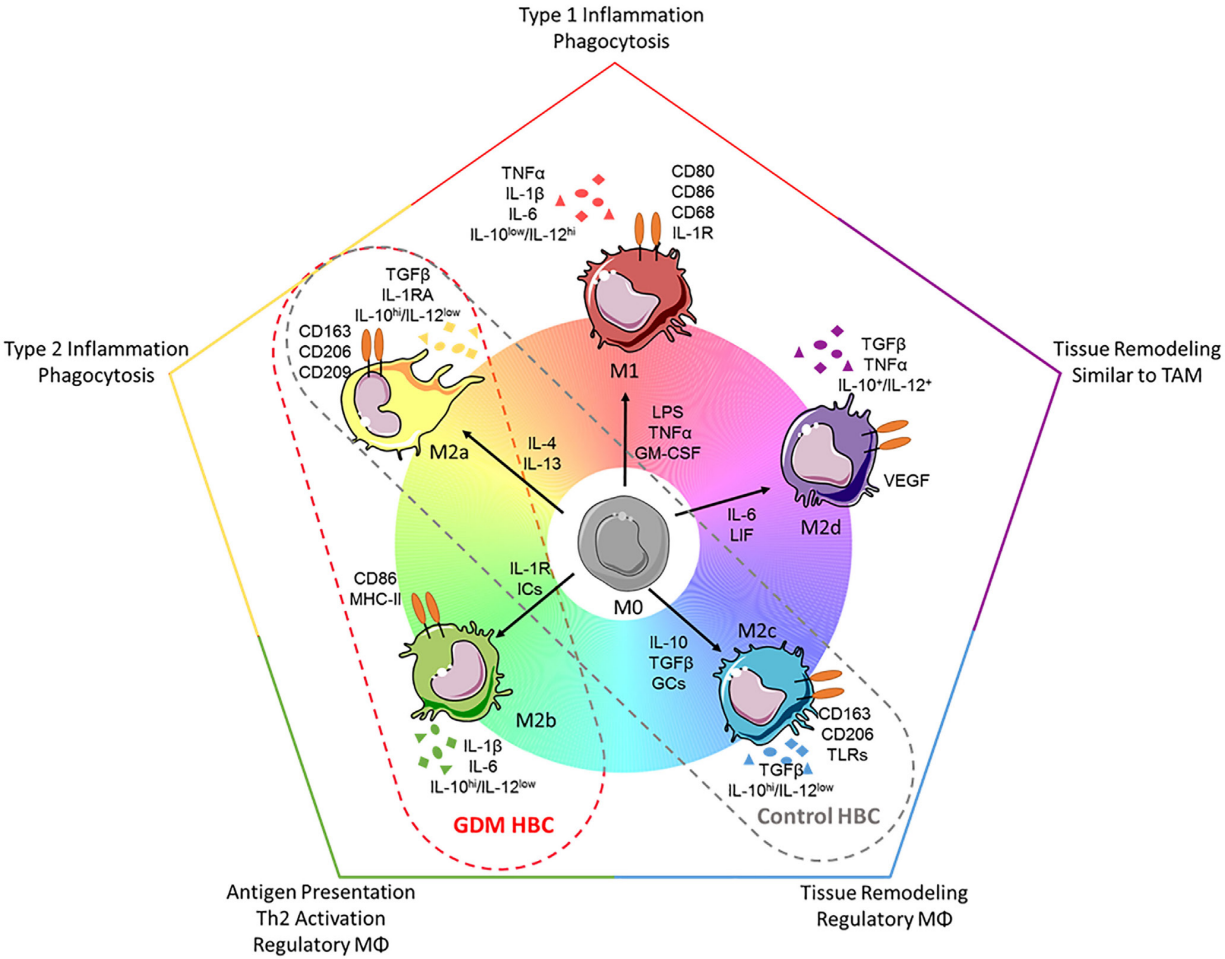
Polarization spectrum of human macrophages and how to apply it to Hofbauer cells (HBC). *Upper panel: current knowledge about macrophages*. Despite the fact that macrophages may be polarized in many intermediate states of the spectrum presented here [adapted from Ref. (11)], generally macrophages are divided into the two categories: M1 (classical/pro-inflammatory) and M2 (alternative/anti-inflammatory). Because of the huge range of macrophage plasticity, the M2 phenotype has been further divided into the subsets M2a, M2b (which shares features of M1), M2c, and M2d macrophages. Each subset can be distinguished looking at (1) induction by certain cytokines, (2) expression of surface markers, (3) release of certain cytokines, or (4) their functionality (phagocytic vs. regulatory or remodeling). *Lower panel: HBCs in the presented study*. In our study, HBCs isolated from control placental tissue displayed features of the M2a and M2c phenotype, e.g., high expression of CD209 and low secretion of interleukin (IL)-6. HBCs isolated from diabetic placenta, on the other hand, displayed some features attributed to M2b phenotype, e.g., elevated (but not significant) expression of the surface marker CD86, and increased (however, also not significant) release of IL-1β and IL-6. Importantly, independent of diabetes during pregnancy, all HBCs in this study were characterized as IL-10^hi^/IL-12^−^, a characteristic separating also M2b cells clearly from M1 polarized macrophages. Abbreviations: ICs, immune complexes; GCs, glucocorticoids; TAMs, tumor-associated macrophages; VEGF, vascular endothelial growth factor. Drawings of macrophages have been adapted from Servier Medical Art (http://www.servier.com/Powerpoint-image-bank), permission is granted under a Creative Commons 3.0 unported license (https://creativecommons.org/licenses/by/3.0/).

The pro-inflammatory, hyperglycemic environment of diabetes was shown to alter macrophage polarization in atherosclerotic plaques (52) and pancreatic islets (53) and *in vitro* models of pre-diabetes (54). Also, GDM induces a pro-inflammatory, hyperglycemic environment in the feto-placental compartment. However, although we investigated a representative set of different M1 and M2 markers and characteristics, we could not observe a trend to increased M1 polarization. What we did observe was even an increase in certain M2 macrophage markers (CD206, CD209), as well as a trend toward increased M1 marker CD86 and increased secretion of IL-1β and IL-6 in GDM macrophages. In fact, M2 macrophages represent at least three subgroups, M2a, M2b, and M2c macrophages (Figure 8). They differ in expression of surface molecules and cytokines, and functions. We have previously described that HBC constitute a mixture of M2a, M2b, and M2c macrophages (25). The observed changes in surface molecules and secreted cytokines would, therefore, indicate a shift toward an increase in M2a population (reflected by enlarged CD206^+^ together with CD209^+^ populations) as well as an increase in M2b populations (reflected by a trend toward increased CD86^+^ population and elevated—though not significant—secretion of IL-1β and IL-6). M2a polarized macrophages indeed play a role in type 2 inflammation. M2b macrophages share a panel of markers with the M1 phenotype, but are considered regulatory macrophages. Whether the shift to more M2a and M2b polarized HBCs may indicate a reduction of M2c polarized HBC is not yet clear. In immune histochemistry, a reduction of CD206 positive cells relative to CD209 positive cells in GDM placenta was observed, hinting that the M2c subset might be reduced; however, in FACS, no difference in the ratio of CD206 to CD209 positive cells was found as in GDM. M2c macrophages particularly play a role in tissue remodeling processes which is assumed as a central function of HBC. Recent results published by our group suggest that HBC regulate placental vascular growth (25). In this study, an assay of endothelial cell activation by macrophage-derived factors was carried out. Endothelial ICAM-1 was increased after stimulation with GDM HBC supernatant, other endothelial adhesion molecules and selectins where not regulated differentially in response to diabetic HBC. Thus, it remains inconclusive if a reduction in the tissue remodeling M2c subset, which also has functional consequences, occurs in GDM placenta.

Using cytochemistry, histochemistry, and FACS we observed—independent of the method—the presence of M1 and M2 markers simultaneously. Similar findings have been previously reported; Young et al. found that almost 98% of HBCs expressed CD163 on their surface, but at the same time CD40 positive cells made up between 5 and 40% of the HBC population in FACS (55). This study also showed CD163 and CD40 staining in immunohistochemistry, which is in line with our findings in total placental tissue by IHC and FACS. In contrast to our findings, Young et al. reported absence of CD11b positive cells on HBCs in both FACS and IHC (55). Using FACS and ICC, for which primary cells had been isolated, we found expression of CD11b. However, within total placental tissue using IHC, we did not observe an immune reaction against CD11b. This could either be due to the use of different antibodies in FACS and IHC or due to loss of the epitope in the tissue after paraffin embedding or inefficient antigen retrieval.

In addition to CD11b positive cells, we also found CD11c-positive cells using ICC and FACS. To the best of our knowledge, little is known about CD11b^+^ and CD11c^+^ macrophages in placenta, but these markers have been well established in adipose tissue macrophages [ATMs (56)]. Zeyda et al. found that ATMs in inflamed adipose tissue expressed surface markers of M2 polarization, but secreted cytokines associated with M1 macrophages (57). This observation could also be the case for HBCs. Bari et al. investigated secretion of soluble CD163 (sCD163), and pro-inflammatory IL-6 and TNFα in gestational diabetes using explants of placenta and adipose tissue from pregnant women and found increased secretion of sCD163 and IL-6 from placenta and adipose tissue explants in GDM (58), pointing toward similarities between HBCs and ATMs. Similarly, we found increased secretion of TNFα and IL-6 from GDM-HBC, but these were not significant. In addition, we found increased (but insignificant) release of M1-associated IL-1β from GDM-HBCs. Challier et al. investigated macrophage subsets in placenta of healthy and obese women. It is known that obesity causes similar changes in placental tissue as GDM (59). They found that mRNA levels of IL-6 and TNFα, and also IL-1 were upregulated in obese placentae (60), supporting our findings.

Recently, Sisino et al. proposed that HBCs switch from an M2 to an M1 phenotype in diabetes during pregnancy (29), which is contradicting our results. However, some differences in study design have to be noted. First, this study did not investigate GDM but pre-gestational type 1 diabetes. These two forms of hyperglycemia are substantially different in terms of onset, treatment, and pathophysiology. They measured only mRNA levels of three markers for M1 (CD68, CCR7, IL-1β) and M2 (CD163, CD209, IL-10) for phenotype characterization in a small number of human subjects. Several studies have suggested that CD68 cannot be considered a marker of M1, but is rather a pan-macrophage marker (61). Our study addressed the question of macrophage polarization in gestational diabetes as opposed to type 1 diabetes, and also used a more integrative approach (surface markers *in vitro* and *ex vivo*, cytokine secretion) which might explain the different findings we present here.

We are aware of limitations of our study. First, our study was conducted in only a small set of subjects. This might be important as we found some potentially relevant, but non-significant differences between control and diabetic macrophages (e.g., IL-1β and IL-6 release). In a larger cohort, inter-individual differences would have been more widely dispersed, thereby reaching greater statistical power. Therefore, we conducted a *post hoc* power analysis and looked into confidence intervals of the multiplex panel and its validation by ELISA. Eventually, we realized that, for the multiplex panel, achieved power was below 50% for the majority of parameters examined. However, as this was a screening approach, we validated certain parameters of interest by conventional ELISA. For these ELISAs, achieved power was >70% for the majority of parameters and 95% confidence intervals were increased compared to multiplex (data not shown). Specifically, for IL-1β and IL-6 ELISAs, a power of 99% was achieved despite the small sample size; thus, it is feasible to state that there is no difference between control and GDM HBCs with respect to these cytokines. As further limitation, we have to point out that the majority of methods employed in this study offer only a descriptive characterization of placental macrophages. We did, however, investigate endothelial cell activation by control and diabetic HBC which can be considered a functional assay.

We consider our pilot-study basic research, and comments on a possible clinical impact might be far-fetched and seem like an over-reach. Also the pleiotropic functions of HBCs are still somewhat enigmatic (40); it has been suggested by various studies that one function of HBCs is to regulate placental vasculo- and angiogenesis (23–25). In GDM, often placental hypervascularization is observed (62, 63), resulting in excessive nutrient transport to the fetus and fetal macrosomia. It would have been tempting to relate the HBC polarization state to this hypervascularization and perinatal outcome; however, it is M2 but not M1 macrophages that have been assigned a pro-angiogenic phenotype (64). As it is hardly possible to state that either GDM-HBCs or control-HBCs are “more M2” polarized than the other, one must not draw the conclusion that GDM-HBCs impact placental vasculature, especially as we did not find differences in endothelial cell activation. Other known functions of HBCs, e.g., vertical transmission of viral and bacterial infections, do not relate to the clinical setting of GDM, making it hard to draw conclusions with regard to clinical impact.

Figure 8 summarizes our findings on surface marker expression and cytokine secretion of control and GDM HBC, and relates them to the common scheme of a continuous spectrum of macrophage polarization (10), gray and red dashed ovals are pointing out where in the spectrum control and GDM HBCs, respectively, might fit in. Similar to findings in ATMs (57, 65), but also supported by other studies working with placental HBCs (26, 27, 55), we suggest that tissue-resident HBCs cells maintain an M2 phenotype in spite of a systemic low-grade pro-inflammatory environment in the mother and fetus. Although initially attributed to a non-physiological response, from an evolutionary point of view, maintenance of the M2 phenotype in HBCs under low inflammatory conditions does make sense. Similar to the adaptation in mammals that allows implantation of the embryo in the mother’s womb early in pregnancy, also in the last trimester of pregnancy there is a need for a regulatory macrophage phenotype rather than a pro-inflammatory macrophage subset, in order to maintain maternal tolerance against an immunologically semi-allogeneic fetus (26). Furthermore, recent studies have demonstrated that the fetal immune system is generally more prone to induce regulatory responses and tissue remodeling than elicit classic activation; this is achieved, e.g., by cross talk of amnion mesenchymal cells and macrophages (66), and fetal dendritic cells and T-cells (67).

Our group recently showed that HBCs are capable of inducing placental endothelial cell migration and tube formation (25) thereby contributing to placental angiogenesis. This is in line with other researchers who found that only M2, but not M1, macrophages are pro-angiogenic (52, 64). Tumor-associated macrophages have been found to have an M2 phenotype and contribute to tumor angiogenesis (68, 69). The feto-placental unit and tumors share certain properties (70): first, their cell mass is built up incredibly fast; second, establishing adequate vasculature is important for growth, nutrient, and energy supply; third, evasion of immune recognition is desired for viability. In cancer patients, maintenance of an M2 macrophage phenotype is detrimental, as a missing M2 to M1 switch aids evading recognition of the tumor by the immune system and at the same time facilitates angiogenesis and, therefore, tumor growth (69). With respect to pregnancy, however, these properties are desired in order to induce tolerance against the fetus and supply it with energy and nutrients. Therefore, it is tempting to speculate that creating progeny might be so important, that macrophages are “kept quiet” in their M2 polarization state—even in low-grade inflammatory states such as GDM. This idea is further corroborated by studies showing that activation of macrophages to a phagocytic rather than regulatory phenotype contributes to miscarriage (71).

The now widely accepted idea that a spectrum of different macrophages might co-exist in the same tissue simultaneously supports our findings and underlines that different macrophage subsets fulfilling different functions might be needed for successful pregnancy outcome. Future research in this direction, both with respect to healthy pregnancies but also obstetric pathologies other than GDM, hopefully will aid to our understanding of the role of these highly diverse placental immune cells.

## Ethics Statement

The study was approved by the institutional ethics committee of the Medical University of Graz (27-265 ex 14/15) and all subjects gave written informed consent.

## Author Contributions

CS and CW conceived the study and designed the experiments. JL established the method for macrophage isolation and characterization prior to this study. CS was responsible for isolating macrophages, performed immune cytochemistry and FACS experiments (the latter with JL), conducted ELISA validation of multiplex results, and endothelial activation assay as well as data analysis. MP carried out the multiplex ELISA-on-bead assay for cytokine detection and analyzed multiplex results. All immune histochemistry stainings and Western Blots were done by SK. UH designed the endothelial activation assay. IL-O evaluated all immune histochemistry and cytochemistry pictures by cell counting. AH, GD, and CW provided supervision during data acquisition and manuscript preparation. CS and CW wrote the manuscript with input from all coauthors.

## Conflict of Interest Statement

The authors declare that the research was conducted in the absence of any commercial or financial relationships that could be construed as a potential conflict of interest.

## Abbreviations

ATMs, adipose tissue macrophages; CD, cluster of differentiation; CM, conditioned medium; EGF, epithelial growth factor; FGF basic, fibroblast growth factor; GDM, gestational diabetes mellitus; GCs, glucocorticoids; GM-CSF, granulocyte macrophage colony stimulating factor; HBCs, Hofbauer cells; ICAM-1, intracellular adhesion molecule 1; ICs, immune complexes; IL, interleukin; IFNγ, interferon γ; MaM, macrophage medium; MCP-1, monocyte chemotactic protein 1; MCP-3, monocyte chemotactic protein 3; MPO, myeloperoxidase; MΦ, macrophage; pAECs, placental arterial endothelial cells; PDGF AB, platelet derived growth factor AB; TAMs, tumor-associated macrophages; TGF α/β, transforming growth factor α/β; TLR, toll-like receptor; TNFα, tumor necrosis factor α; VCAM-1, vascular adhesion molecule 1; VE-cadherin, vascular endothelial cadherin; VEGF, vascular endothelial growth factor.
