# Supplementary material for: Human Placental Hofbauer Cells Maintain an Anti-inflammatory M2 Phenotype despite the Presence of Gestational Diabetes Mellitus
Source: Front Immunol. 2017 Jul 31;8:888. doi: 10.3389/fimmu.2017.00888 (PMC5534476; doi:10.3389/fimmu.2017.00888)
Supplement: Supplementary file 1 [file data_sheet_1.docx]

**Supplemental Material for**

Human placental Hofbauer Cells maintain an anti-inflammatory M2 phenotype despite the presence of Gestational Diabetes Mellitus

**Carolin Schliefsteiner^1^, Miriam Peinhaupt^3^, Susanne Kopp^1^, Jelena Lögl^1,2^, Ursula Hiden^1^, Akos Heinemann^3^, Gernot Desoye^1^, Christian Wadsack^1,*^**

^1^Perinatal Research Laboratory, Department of Obstetrics and Gynecology, Medical University of Graz; Graz, Austria

^2^Department of Embryology and Histology, Medical University of Graz; Graz, Austria

^3^Department of Experimental and Clinical Pharmacology, Medical University of Graz; Graz, Austria

*** Correspondence:**

Christian Wadsack, PhD

christian.wadsack@medunigraz.at


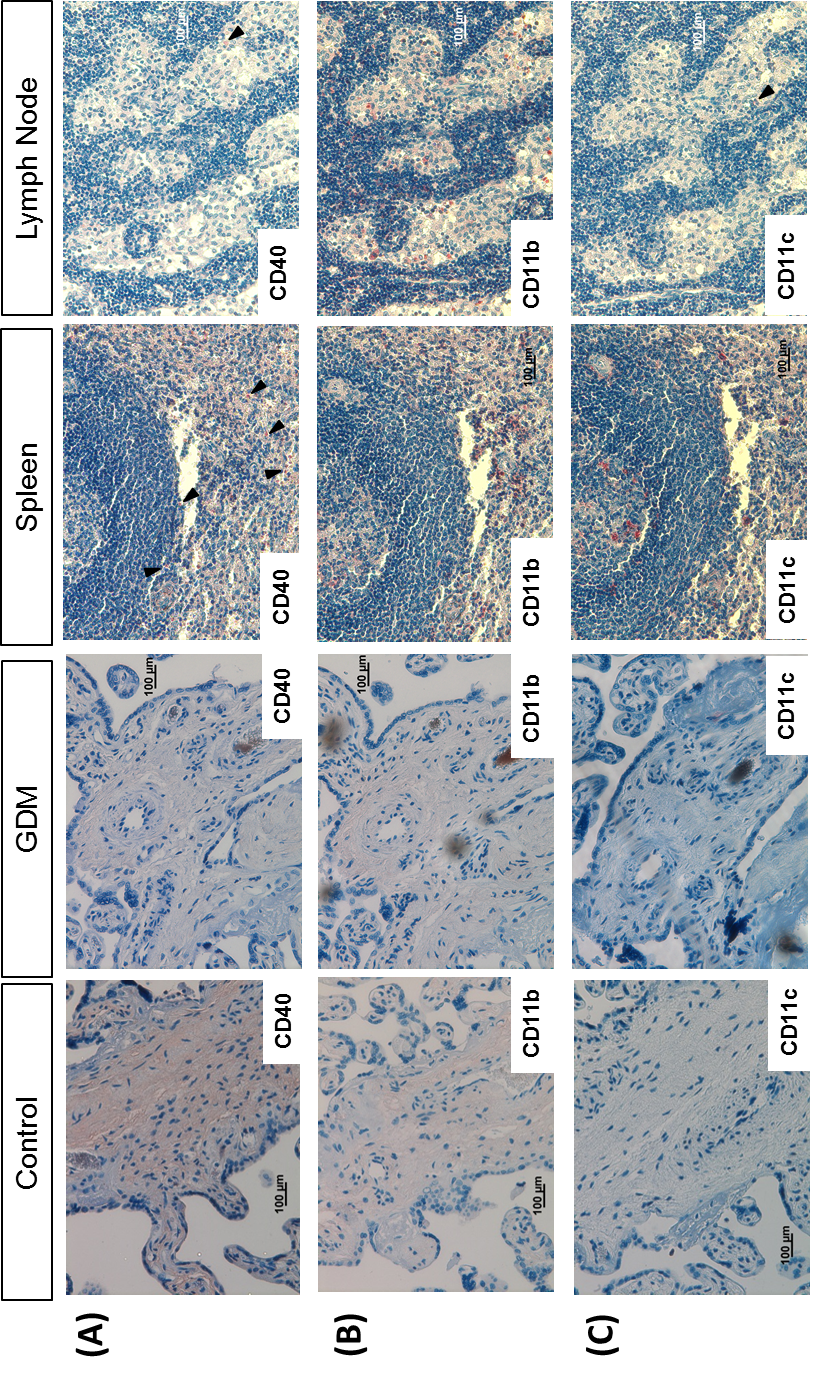


Supplemental Figure 1 Immune histochemistry staining of additional M1 markers in placental tissue. A: Staining against CD40 in control placenta (far left), and GDM placenta (center left); spleen (center right) and lymph node (far right) served as positive controls. B: Staining against CD11b in control placenta (far left), and GDM placenta (center left); spleen (center right) and lymph node (far right) served as positive controls. C: Staining against CD11c in control placenta (far left), and GDM placenta (center left); spleen (center right) and lymph node (far right) served as positive controls. Black arrowheads indicate faint yet positive staining against respective markers; in Control and GDM placenta no specific staining against HBCs in villous stroma was observed. Images representative of four independent stainings per group are shown.

**Supplemental Table 1 Release of Cytokines and Growth Factors from Control and GDM-HBC as measured by Multiplex Array.** All 23 parameters measured in the ELISA-on-bead approach are listed, physiological relevance (pro-/anti-inflammatory action) is provided in the second column. All concentrations are provided as pg/mg of total protein, mean±SD, significance was calculated by Kruskal-Wallis-Test (ANOVA on ranks); † p≤0.09, # p≤0.05, ¶ p≤0.01. Bold print indicates factors that were validated by conventional ELISA.

|  |  | **Control (n=5)** | | | | | | | | | | | **GDM (n=6)** | | | | | | | | | | |
| --- | --- | --- | --- | --- | --- | --- | --- | --- | --- | --- | --- | --- | --- | --- | --- | --- | --- | --- | --- | --- | --- | --- | --- |
| **Cytokines/ Growth factors** | **Physiological Relevance** | **24h** | | | **48h** | | | **72h** | | | **96h** | | **24h** | | | **48h** | | | **72h** | | | **96h** | |
| IL-1α | pro-inflammatory, M1 | N/A | | 0.6±1.1 | | | 0.7±1.2 | | | 2.2±3.2 | | | 1.22±0.8 | | 2.48±0.3 | | | 1.99±0.3 | | | 1.96±0.2 | | |
| **IL-1β** | **pro-inflammatory, apoptotic** | **1.4±0.7** | | **5.8±9.3** | | | **5.4±6.0** | | | **10.4±8.3** | | | **8.2±8.0** | | **14.9±15.9** | | | **15.6±19.8** | | | **14.6±18.8** | | |
| **IL-1RA** | **anti-inflammatory** | **2776.0±**  **1279.1** | | **3344.2±**  **1751.5** | | | **4216.8±**  **554.9** | | | **4449.5±**  **526.5** | | | **2307.8±**  **475.4** | | **3534.2±**  **953.7** | | | **4138.5±**  **821.9** | | | **4058.8±**  **1438.0** | | |
| IL-4 | anti-inflammatory, M2 | N/A | | N/A | | | N/A | | | N/A | | | N/A | | N/A | | | N/A | | | N/A | | |
| **Il-6** | **pro- /anti-inflammatory** | **101.9±**  **50.8** | | **520.9±**  **822.8** | | | **645.6±**  **873.4** | | | **905.0±**  **1139.4** | | | **508.2±**  **548.5** | | **897.6±**  **956.4** | | | **816.2±**  **961.5** | | | **622.5±**  **740.4** | | |
| **IL-8** | **NP attraction, angiogenesis** | **3076.8±**  **422.0** | | **3039.5±**  **606.0^#^** | | | **3706.2±**  **312.2** | | | **3841.7±**  **709.0** | | | **2889.1±**  **434.9** | | **4161.8±**  **369.4^#^** | | | **4003.8±**  **604.1** | | | **4010.3±**  **395.4** | | |
| **IL-10** | **anti-inflammatory, M2** | **2.2±1.5** | | **3.0±4.0** | | | **2.0±2.2** | | | **1.5±1.8** | | | **4.4±2.9** | | **5.5±4.9** | | | **2.9±3.4** | | | **1.9±1.9** | | |
| IL-12p70 | anti-angiogenic, M1 | 0.2±0.3 | | 0.3±0.3 | | | 0.3±0.3 | | | 0.2±0.3 | | | 0.4±0.3 | | 0.5±0.4 | | | 0.3±0.4 | | | 0.3±0.4 | | |
| **Il-13** | **anti-inflammatory, M2** | **48.7±23.5** | | **63.7±48.6** | | | **85.7±32.1** | | | **83.6±46.8** | | | **59.5±26.8** | | **97.7±47.2** | | | **85.7±51.1** | | | **103.6±64.0** | | |
| EGF | proliferation, cell survival | N/A | | N/A | | | N/A | | | N/A | | | N/A | | N/A | | | N/A | | | N/A | | |
| FGF basic | angiogenesis, wound healing | 20.8±27.5 | | 18.4±23.1 | | | 30.3±17.5 | | | 37.6±27.0 | | | 30.0±16.1 | | 47.8±26.0 | | | 36.8±17.5 | | | 26.8±31.2 | | |
| PDGF AB | angiogenesis, mitogen | 1318.0±  821.9 | | 1981.3±  1166.2 | | | 2423.3±  1266.0 | | | 2386.8±  1188.3 | | | 1403.1±  562.3 | | 2557.7±  1365.2 | | | 1587.9±  1519.3 | | | 2952.5±  1755.1 | | |
| **VEGF** | **angiogenesis, vasculogenesis** | **449.2±**  **147.1^#^** | | **667.6±**  **387.6^†^** | | | **848.9±**  **305.5** | | | **792.8±**  **435.7** | | | **663.1±**  **96.9^#^** | | **1028.3±**  **188.7^†^** | | | **988.9±**  **313.7** | | | **933.2±**  **343.1** | | |
| TGFα | mitogen, proliferation, | 45.1±28.8 | | 61.4±30.6**^†^** | | | 123.3±  107.9 | | | 194.0±  222.6 | | | 62.0±23.6 | | 93.9±21.9**^†^** | | | 64.7±46.4 | | | 218.0±  147.5 | | |
| **ICAM-1** | **Adhesion, pro-inflammatory** | **844.1±**  **238.4** | | **1002.4±**  **344.4^¶^** | | | **1094.6±**  **97.1** | | | **1275.7±**  **121.5** | | | **868.6±**  **163.2** | | **1904.2±**  **687.4^¶^** | | | **1152.5±**  **520.1** | | | **1260.1±**  **352.2** | | |
| VCAM-1 | Adhesion, pro-inflammatory | 144.3±  37.3 | | 162.5±  57.9 | | | 175.9±  30.4 | | | 171.5±2.7 | | | 120.4±  28.1 | | 180.3±  26.4 | | | 156.9±  54.2 | | | 165.62±  40.3 | | |
| **MCP-1** | **Adhesion, pro-inflammatory,** | **511.7±**  **236.7** | | **298.9±**  **119.4** | | | **730.3±**  **61.5** | | | **558.2±**  **142.6** | | | **569.3±**  **85.7** | | **625.5±**  **263.1** | | | **589.4±**  **285.6** | | | **644.8±**  **247.4** | | |
| MCP-3 | monocyte attraction, | 89.6±  110.4 | | 136.3±  107.8 | | | 127.7±  99.6 | | | 133.9±89.8 | | | 67.2±66.9 | | 173.8±  156.8 | | | 90.5±  128.9 | | | 158.7±  180.0 | | |
| GM-CSF | Neutrophil attraction,  MΦ differentiation | 54.5±42.7 | | 88.2±76.7 | | | 80.5±52.7 | | | 115.6±  65.6† | | | 39.1±11.1 | | 60.1±32.9 | | | 44.8±30.0 | | | 44.0±  28.0† | | |
| **TNFα** | **pro-inflammatory, M1** | **7.0±4.5** | | **14.9±28.6** | | | **5.6±8.9** | | | **6.2±8.0** | | | **28.1±27.5** | | **10.7±13.4** | | | **6.3±8.5** | | | **2.7±3.0** | | |
| INFɣ | M1, pro-inflammatory | N/A | | N/A | | | N/A | | | N/A | | | N/A | | N/A | | | N/A | | | N/A | | |
| Leptin | energy homeostasis | 666.5±  376.2 | | 933.0±  165.0**^†^** | | | 991.5±  233.3 | | | 938.5±  510.1 | | | 855.0±  108.2 | | 1181.28±  195.8**^†^** | | | 794.5±  416.2 | | | 1145.4±  287.5 | | |
| MPO | ROS production | 0.6±0.2**^#^** | | 0.7±0.3 | | | 0.7±0.2 | | | 0.8±0.3 | | | 0.4±0.1**^#^** | | 0.6±0.2 | | | 0.5±0.1 | | | 0.6±0.1 | | |
| **†** p≤0.09, # p≤0.05, ¶ p≤0.01 | | |  | | |  | | |  | | |  | |  | | |  | | |  | | |  |
